# Prediction of thermophysical properties of R-454B based on molecular dynamic simulation and SAFT-based equation of state

**DOI:** 10.1038/s41598-025-03928-2

**Published:** 2025-06-05

**Authors:** Farag M. A. Altalbawy, Fadhil Faez Sead, Ramdevsinh Jhala, T. Ramachandran, Junainah Abd Hamid, Aman Shankhyan, A. Karthikeyan, Dhirendra Nath Thatoi

**Affiliations:** 1 Department of Chemistry, University College of Duba, University of Tabuk, Tabuk, Saudi Arabia; 2 Department of Dentistry, College of Dentistry, The Islamic University, Najaf, Iraq; 3 Department of Dentistry, College of Dentistry, The Islamic University of Al Diwaniyah, Al Diwaniyah, Iraq; 4 Department of Dentistry, College of Dentistry, The Islamic University of Babylon, Babylon, Iraq; 5 Marwadi University Research Center, Department of Mechanical Engineering, Faculty of Engineering & Technology, Marwadi University, Rajkot, Gujarat 360003 India; 6 Department of Mechanical Engineering, School of Engineering and Technology, JAIN (Deemed to Be University), Bangalore, Karnataka India; 7 Management and Science University, Shah Alam, Selangor Malaysia; 8 Centre for Research Impact & Outcome, Chitkara University Institute of Engineering and Technology, Chitkara University, Rajpura, 140401 Punjab India; 9 Department of Mechanical Engineering, Sathyabama Institute of Science and Technology, Chennai, Tamil Nadu India; 10 Department of Mechanical Engineering, Siksha ‘O’ Anusandhan (Deemed to Be University), Bhubaneswar, Odisha 751030 India

**Keywords:** Molecular simulations, Heat capacity, PC-SAFT, R-454B, Refrigerant, Speed of sound, Chemical engineering, Computational methods

## Abstract

R-454B is an excellent choice for refrigeration systems due to its environmentally friendly profile. In this study, the thermophysical properties of R-454B refrigerant are predicted using molecular dynamics (MD) simulations coupled with a SAFT-based equation of state (EoS). Since experimental data on the thermophysical properties of R-454B are generally scarce in technical applications, exploring these properties is essential. In this work, the COMPASS force field is employed to develop the MD simulations. The saturated density, vapor pressure, and isobaric heat capacity of R-454B were simulated. The average ARD% for the isobaric heat capacity was approximately 7.66% over the temperature range of 273.15–303.15 K. The PC-SAFT equation of state (EoS) was coupled with MD simulation to predict the thermodynamic properties of R-454B across a broad range of pressures and temperatures. In this regard, the PC-SAFT model parameters were adjusted using the simulated saturated liquid density and vapor pressure data. The obtained PC-SAFT model parameters were utilized to predict the speed of sound, specific heat capacity, and Joule–Thomson coefficient of R-454B. The results indicate that the proposed model can satisfactorily predict the vapor and liquid thermophysical properties of R-454B. This methodology can be employed to estimate second-order derivative thermodynamic properties of novel refrigerants prior to synthesis, potentially reducing the costs and time associated with experimental development.

## Introduction

Thermophysical properties of refrigerants are essential for designing and optimizing refrigeration systems. These properties determine the refrigerant’s behavior under various operating conditions and significantly influence system efficiency, safety, and environmental impact. The most critical properties vary depending on the specific application and system design. Molecular dynamics (MD) simulations have emerged as a valuable tool for investigating these thermophysical properties. MD simulations offer a microscopic perspective, complementing experimental data and providing insights into the molecular mechanisms underlying refrigerant behavior. They can be used to predict and analyze a wide range of properties, including density, critical properties, enthalpy, internal energy, entropy, specific heat capacity, latent heat of vaporization, viscosity, thermal conductivity, diffusion coefficients, surface tension, and the liquid–vapor coexistence curve. Additionally, simulations can be employed to estimate the thermophysical properties of novel refrigerants prior to synthesis, potentially reducing the costs and time associated with experimental development. MD can analyze the behavior of refrigerant mixtures, providing insights into how their properties depend on composition. These simulations are valuable for developing new refrigerants with tailored thermophysical properties and low environmental impact, as well as for enhancing the design and efficiency of refrigeration systems by understanding molecular-level behavior. Additionally, MD allows exploration of conditions that are challenging or impossible to study experimentally, such as extremely high pressures or low temperatures. However, the accuracy of simulation results heavily relies on the quality of the force field employed. A major limitation of MD is its computational expense, often requiring long simulation times, especially for large systems.

R-454B is a blend comprising 68.9 wt% R-32 and 31.1 wt% R-1234yf. In recent years, R-454B has been used as an alternative to refrigerant R-410A. It has a global warming potential (GWP) of 466, which is approximately 78% lower than that of R-410A, making it a more environmentally friendly option^1^. R-454B is often compatible with existing R-134a systems, reducing the need for significant retrofits, although minor modifications might still be required. Primarily used in automotive air conditioning, it also finds applications in other refrigeration sectors where a low-GWP alternative to R-134a is desired. Its exact formulation is typically proprietary, but its general properties are well-documented. As a zeotropic mixture, modeling and predicting its performance can be more complex than for pure refrigerants. Simulating R-454B requires advanced expertise in force field development and MD simulation. Achieving accurate results often depends on access to experimental data for the specific blend, including interaction data between components, as well as a thorough understanding of the inherent limitations and assumptions.

Alam and Jeong utilized the MD simulation to study the condensation process of R143a, R600, and R134a^2^. As well, they estimated the thermodynamic properties of R448A and R449A using MD simulations^3^. X. Liu et al. used MD simulation to simulate the vapor condensation processes of R1243zf, R1234ze and R1234yf^4^. The isobaric heat capacities and densities of R1234ze, R1243zf, and R1234yf were simulated and compared to experimental data. G. Raabe utilized MD simulations to simulate the VLE in binary mixtures of the R-1234ze and R-1234yf with CO_2_, and R-32^5^. She used a combining rule to determine the Lennard–Jones parameter between atoms. So, the obtained results are purely predictive. She suggested a force field for MD simulation of R-1234yf^6^. Fouad and Alasiri predicted the liquid densities, viscosity, and self-diffusion coefficients of R-1224yd, R-152a, R-32, R134a, R-1234ze, R-1234yf, R-1233zd, and R-1336mzz by using MD simulation^7^. Their results demonstrated that transport properties are strongly dependent on the refrigerant structure, size, and long-range interactions. Wang et al. studied the thermophysical properties of R1233zd(E) using MD^8^. They used the MD simulation method to study the heat capacity and density of R1233zd(E). Y. Li et al. utilized the MD simulations to study the VLE and interfacial properties of some refrigerants and their blends^9^. Their results show that a combination of MD and equation of state calculations can provide accurate results for the design of new blends of refrigerants.

The EoSs were widely used to predict the phase equilibria and thermophysical properties of fluids^10–21^. M. Doubek and V. Vacek utilized the PC-SAFT, and SAFT-BACK models to estimate the speed of sound of some refrigerants^22^. K. Parvaneh et al. used the QC-PC-SAFT EoS to estimate the phase behavior of IL + refrigerant systems^23^. Swaminathan and Visco used the SAFT-VR (variable range) EoS to study the phase equilibrium of pure refrigerants^24^. Polishuk et al. correlated and predicted various thermodynamic properties of 11 refrigerants using the SAFT + Cubic and PC-SAFT^25^. As well, they utilized the CP-PC-SAFT to estimate the heat capacities and speed of sound of refrigerants^26^. Recently, Abed et al. utilized the SAFT-VR Morse^12,15,27^ to estimate the derivative thermodynamic properties of refrigerants^28^.

The primary challenges in modeling R-454B refrigerant with thermodynamic models, such as equations of state (EoS), arise from its non-ideal behavior as a blend and the inherent complexity of accurately representing its phase equilibrium and thermodynamic properties across a broad range of operating conditions. As a refrigerant blend, R-454B poses greater modeling difficulties compared to single-component refrigerants. Specifically, the intermolecular interactions within the blend must be accurately represented by the EoS, as these interactions significantly influence properties such as vapor pressure, density, enthalpy, heat capacity, and speed of sound. Many commonly used EoS models (e.g., cubic-based and SAFT-based models) may lack the accuracy required for R-454B, particularly at extreme temperatures and pressures. These equations often necessitate modifications or the implementation of more sophisticated mixing rules to accurately represent the behavior of fluorinated refrigerants, such as those present in R-454B. Furthermore, accurate EoS models rely on reliable experimental data for parameter fitting and validation. However, the limited availability of experimental data for R-454B, especially across various compositions and conditions, can hinder the development and validation of accurate models. Consequently, accurate modeling of R-454B demands careful selection or development of an appropriate thermodynamic model, accurate representation of the blend’s mixing behavior, ensuring sufficient experimental data for parameter fitting and validation, and balancing accuracy with computational cost.

R-454B is a relatively new refrigerant blend with a scarcity of published experimental thermodynamic data. Consequently, developing accurate EoS models or adapting existing models for R-454B necessitates the creation of novel parameterizations and validation approaches that lack extensive documentation. To address this gap, this work presents the development of a predictive SAFT-based EoS. The primary novelty of the proposed model lies in the parameterization of the PC-SAFT EoS utilizing MD simulations. Specifically, first-order derivative thermodynamic properties, including vapor pressure and saturated liquid density of R-454B, were obtained from MD simulations, and these data were then used to adjust the PC-SAFT model parameters. Notably, second-order derivative thermodynamic properties, such as speed of sound, heat capacity, and the Joule–Thomson coefficient, were predicted without the use of any additional adjustable parameters. Thus, the model parameters were determined independently of experimental data, addressing a significant limitation of traditional thermodynamic models (such as SAFT-based EoS) that often suffer from a scarcity of experimental data for parameterization. This methodology effectively overcomes the data gap challenge for this new refrigerant blend. Furthermore, a simplification was introduced by treating the R-454B blend as a pseudo-component within the PC-SAFT EoS parameterization, thereby avoiding the need for complex mixing rules. While the blend was treated as a pseudo-component in the PC-SAFT EoS parameterization, two distinct particle types were considered in the MD simulations.

This work aims to develop a predictive EoS capable of accurately estimating the thermodynamic properties of R-454B refrigerant without relying on experimental data. While MD simulations offer valuable insights, their computational demands are significantly higher than those of SAFT-based EoS models. Therefore, this study leverages MD simulation results for R-454B to optimize PC-SAFT parameters, enabling the subsequent prediction of properties such as speed of sound, specific heat capacity, and the Joule–Thomson coefficient using the PC-SAFT EoS. The proposed method provides a valuable tool for predicting the thermophysical properties of R-454B in the absence of experimental data and holds promise for future applications in the design of novel refrigerants tailored to specific processes.

## Molecular dynamic simulation

**Fig. 1:**
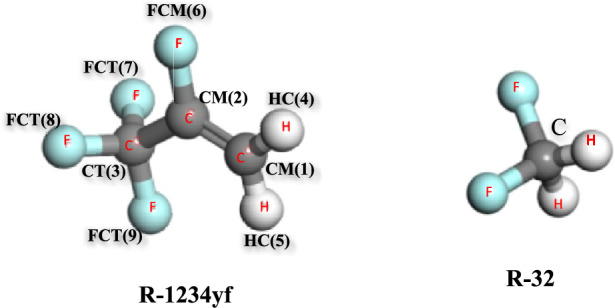
Structures of the 2,3,3,3-tetrafluoro-1-propene (R-1234yf) and R-32.

Simulating R-454B is advanced work requiring expertise in both force field development and MD simulation. It is highly unlikely to achieve accurate results without access to experimental data for the specific blend, including interaction data between the components, and a detailed understanding of the limitations and assumptions involved. However, MD simulation is one of the best methods for estimating the thermophysical properties of a fluid in the absence of experimental data. In Fig. 1, the molecular structures of R-1234yf and R-32 have been depicted.

The force field in this study has been developed based on the COMPASS II force field as follows^29,30^:

1 $$\begin{aligned}{U}_{Conf} = &\, \sum_{bond}{k}_{r}{(r-{r}_{0})}^{2}+\sum_{angles}{k}_{\theta }{(\theta -{\theta }_{0})}^{2}+\sum_{dihedral}{k}_{\chi }\left[1+\text{cos}(n\chi -\delta )\right]\\&+\sum_{i}\sum_{j>i}\left\{4{\varepsilon }_{ij}\left[{\left(\frac{{\sigma }_{ij}}{{r}_{ij}}\right)}^{12}-{\left(\frac{{\sigma }_{ij}}{{r}_{ij}}\right)}^{6}\right]+\frac{1}{4\pi {\varepsilon }_{0}}\frac{{q}_{i}{q}_{j}}{{r}_{ij}}\right\}\end{aligned}$$

The most crucial aspect is accurately representing the interactions between the different molecules in the mixture. Standard combining rules (like Lorentz-Berthelot) might be a starting point, but they might not be sufficiently accurate. The interactions between molecules have been modeled by the Lennard–Jones (LJ) potential. The electrostatic interactions have been considered by using fixed partial charges on molecules^31^. The Lorentz-Berthelot combining rule is utilized to obtain all unlike atoms parameters of LJ potential. The harmonic terms for bond stretching are used to describe the intramolecular potential energy^5,31^. The *r*_*0*_ is average nominal bond lengths and *θ*_*0*_ is bond angles. The aforementioned parameters were obtained from simulations of the bond stretching by perturbing the bond lengths using their equilibrium value^5,31^. All simulations were calculated using B3LYP/DGDZVP level of theory. In this regard the Gaussian 03 package is utilized. Details of the parametrization are summarized in^32^.

R-454B is a blend of 68.9%wt R-32, and 31.1%wt R-1234yf. Therefore, the force field parameters of R-32 and R-1234yf must be estimated to develop the MD simulations. In the case of R-32, the filed force parameters for the F–C-F angle and C-F bond have been taken from the fluoropropene force field similar to previous works^5,32^. In Tables 1 and 2 the force field parameters of R-32 and R-1234yf have been reported.

**Table 1:** Force field parameters for the R-32.

Atom	σ($\dot{A}$)	$\varepsilon /k$(K)	q(e)
C	3.15	54.6	0.43960
F	2.94	44.0	− 0.26138
H2	2.229	7.9	0.04158
Bond	k_r_ (kJ/mol.$\dot{A}$^2^)	r_0_ ($\dot{A}$)	
C-F	1544.6	1.369	
C-H	1472.89	1.094	
Angle	k $\theta$ (kJ/mol.$rad$^2^)	${\theta }_{0}(deg)$	
H-C-H	146.54	113.6	
F–C-F	367.61	108.7	
H-C-F	249.92	108.6

**Table 2:** Force field parameters for the R-1234yf.

Atom	σ($\dot{A}$)	$\varepsilon /k$(K)	q(e)
CM(1)	3.4	49.31	− 0.41911
CM(2)	3.4	49.31	0.19743
CT(3)	3.4	37.39	0.63064
FCM(6)	2.9	28.40	− 0.18254
FCT(7,8,9)	2.94	28.40	− 0.21196
HC(4,5)	2.65	7.90	0.20473
Bond	k_r_ (kJ/mol.$\dot{A}$^2^)	r_0_ ($\dot{A}$)	
CM=CM	2831.7	1.331	
CM–CT	1328.84	1.511	
CT–FCT	1544.61	1.353	
CM–HC	1627.07	1.086	
CM–FCM	1864.73	1.330	
Angle	k$\theta$ (kJ/mol.$rad$^2^)	${\theta }_{0}(deg)$	
HC–CM=CM	152.09	120.6	
FCT–CT–FCT	367.61	107.5	
CM–CT–FCT	313.17	111.3	
HC–CM–CT	135.27	115.1	
HC–CM–HC	122.63	118.7	
CM=CM-FCM	211.38	122.6	
FCM–CM–FCM	357.23	112.6	
FCM–CM–CT	319.57	112.6	
CM=CM–CT	209.70	124.1	
Dihedral	${k}_{\chi }$	n	$\delta (deg)$
X–CM=CM–X	27.84	2	180
HC–CM–CT–FCT	0.745	3	0
FCM–CM–CT–FCT	1.0445	3	0
CM=CM–CT–FCT	0.5951	3	180

The Lorentz-Berthelot combining rules (Eqs. 2 and 3) have been used to estimate the LJ parameters in mixtures:

2 $${\varepsilon }_{ij}=\sqrt{{\varepsilon }_{i}{\varepsilon }_{j}}$$

3 $${\sigma }_{ij}=\frac{{\sigma }_{i}+{\sigma }_{j}}{2}$$

In this study, VLE of the binary mixtures has been calculated using the Monte Carlo (MC) Gibbs ensemble. In this regard the isothermal-isobaric (NPT) ensemble Gibbs ensemble is utilized^31^. Each ensemble consisted of 256 molecules. The number of molecules of both components in R-454B is considered based on the blend definition. The Ewald sum method is utilized for the electrostatic interactions. The cutoff radius for the LJ is set to 12 Å^31^. The simulations have been equilibrated for 100,000 cycles. The production runs of 50,000 to 100,000 cycles have been considered, and each cycle consisted of 256 attempted moves. The moves have been defined at random and with a fixed probability. The ratios for attempted moves have been considered about 65% intrabox moves, 35% interbox molecular transfer moves, and 0.5% volume moves^31^. In the next section the MD simulation results have been described.

## Results and discussion

### MD simulations

A cubic cell with periodic boundary conditions in all directions is built for R-454B in the temperature range of 273.15–348.15 K. The total number of particles employed in molecular simulations has a significant effect on the accuracy and reliability of the results, impacting various aspects of the simulation. More particles usually lead to better sampling of the phase space. This is crucial for accurate averages of properties like energy, pressure, and density. With more particles, the fluctuations in these properties become smaller, leading to more reliable estimates. This is particularly important when dealing with systems exhibiting large fluctuations, like liquids or gases. A larger system size can better represent the bulk behavior of the system. However, simulating a larger system requires more computational resources, in terms of memory and processing power. The computational time increases proportionally with the number of particles, often significantly. Extremely large systems can lead to numerical instability or difficulties in solving the equations of motion, especially if the system’s interactions are complex. The optimal number of particles depends heavily on the system being modeled. In this work scaling relations has been used to find the appropriate number of molecules in simulation box. Using scaling relations to find the optimum number of molecules in a molecular dynamics MD simulation box is a crucial step in ensuring efficient and accurate results. There isn’t a single “optimal” number, as it depends on the system. The key scaling relations relate the system properties to the number of particles (N), the volume (V), and the temperature (T). These are often derived from thermodynamic relations and the nature of the interactions between particles. The results show that simulations using 256 molecules in the simulation box produce suitable results. Based on the molar composition of R-32 and R-1234yf in R-454B blend, 212 molecules for R-32 and 44 molecules for R-1234yf are considered. The cell is optimized, and the MD-NVT simulations are performed by using the Materials Studio^33^ to calculate the saturated vapor pressures.

The key to determining the initial simulation box size for NVT MD simulations to calculate saturated vapor pressure and density is balancing theoretical calculations with practical simulation considerations. Below are the steps to determine the initial MD simulation box size:

- The system and phase should be defined, including temperature and pressure.
- The number of molecules should be estimated based on the desired vapor density. Available literature values or correlations were utilized to estimate the density of the vapor phase at the target temperature.
- The appropriate volume should be calculated based on the estimated density and molecular count (V = NMw/ρNA). Then this volume should be converted into a cubic box (L = ${V}^{1/3}$).
- The volume should be adjusted to accommodate intermolecular interactions and avoid edge effects from periodic boundaries.
- An initial configuration should be created, and equilibration should be run to ensure proper thermodynamic behavior.

Multiple preliminary simulations should be conducted using different box sizes. During these runs, the density profile within the box and the pressure throughout the simulation must be monitored. The results are then used to adjust the box size, and the simulation is rerun iteratively until the density profile and pressure align with the expected values. The equilibration stimulation is first performed for 2.5 ns, and then a production run is conducted for another 2.5 ns. In this work, 1 fs time step is considered. Standard deviations (SD) of all ensemble averages were determined by the standard block average technique^34^. SD of all ensemble averages were calculated by dividing the production runs into 10 blocks. The SD in vapor pressure, heat capacity, and saturated liquid density was estimated from the NVT run after completion. The expanded uncertainties in vapor pressure, saturated liquid density, and heat capacity, with a 95% level of confidence, were obtained by multiplying SD by a coverage factor of 2. The vapor pressure of R-454B at seven temperatures between 273.15 and 348.15 K has been simulated. The results have been compared to the NIST Standard Reference Database (REFPROP)^35^ and the ARD% has been reported in Table 3. It must be noted that, REFPROP cannot serve as a definitive benchmark for predicting the thermophysical properties of the R-454B blend. While REFPROP correlations were used as a suitable reference point for validating the MD simulation results, the lack of experimental data for the blend means that REFPROP’s predictions for the blend are likely not entirely accurate, and thus, the validation is not fully definitive. The lack of experimental data means the validation will necessarily be weaker and more limited than if experimental data were available.

**Table 3:** Simulated vapor pressure of R-454B.

T(K)	P^sat^(MPa)		ARD%
	Simulated	REFPROP^35^	
273.15	0.756 (± 0.011)	0.776	2.58
283.15	1.023 (± 0.014)	1.053	2.85
293.15	1.378 (± 0.010)	1.398	1.43
303.15	1.8 (± 0.013)	1.822	1.21
313.15	2.307 (± 0.009)	2.335	1.20
323.15	2.917 (± 0.017)	2.951	1.15
333.15	3.637 (± 0.012)	3.684	1.28
343.15	4.445 (± 0.017)	4.552	2.35
345.15	4.642 (± 0.023)	4.745	2.17
348.15	4.956 (± 0.021)	5.044	1.74
Average error			**1.79**

Significant values are in bold.

**Fig. 2:**
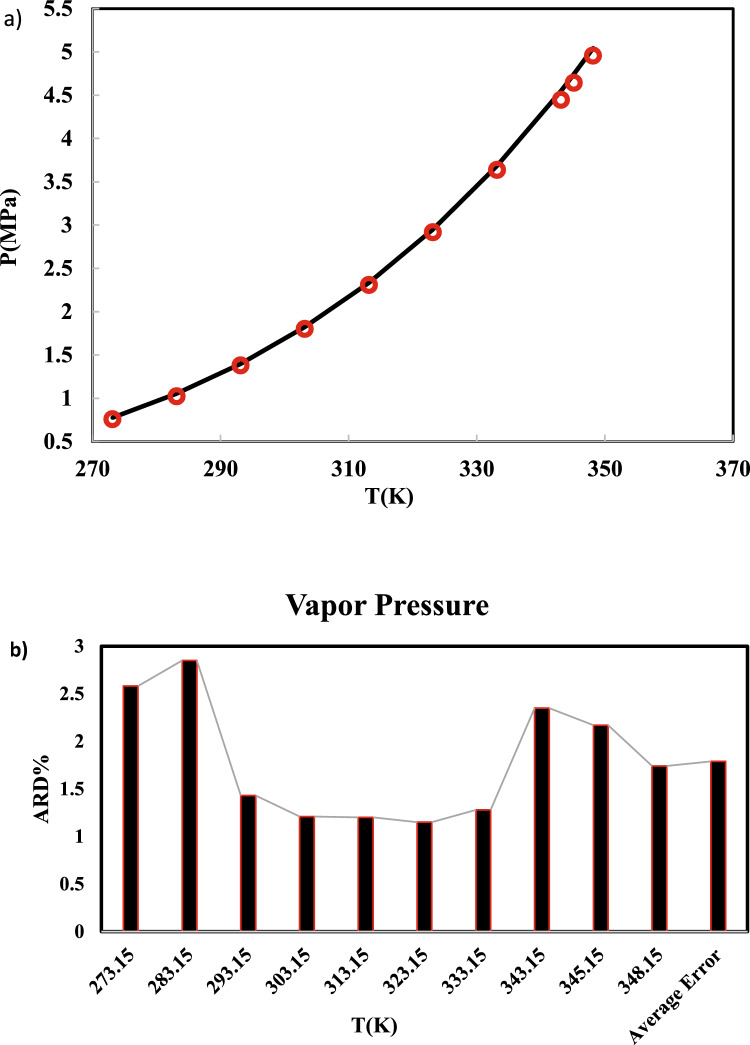
(**a**) Computed vapor pressures of R-454B (Symbols) and REFPROP (line). (**b**) Error bar of vapor pressure.

In Fig. 2, the simulated vapor pressure has been compared to REFPROP data.

As shown in Table 3 and Fig. 2a, the average relative deviation (ARD%) between simulated and RFPROP results is 1.79%. As well, the error bar of vapor pressure has been depicted in Fig. 2b. It is indicated that the proposed simulation and force field can simulate the vapor pressure of R-454B, satisfactory.

**Fig. 3:**
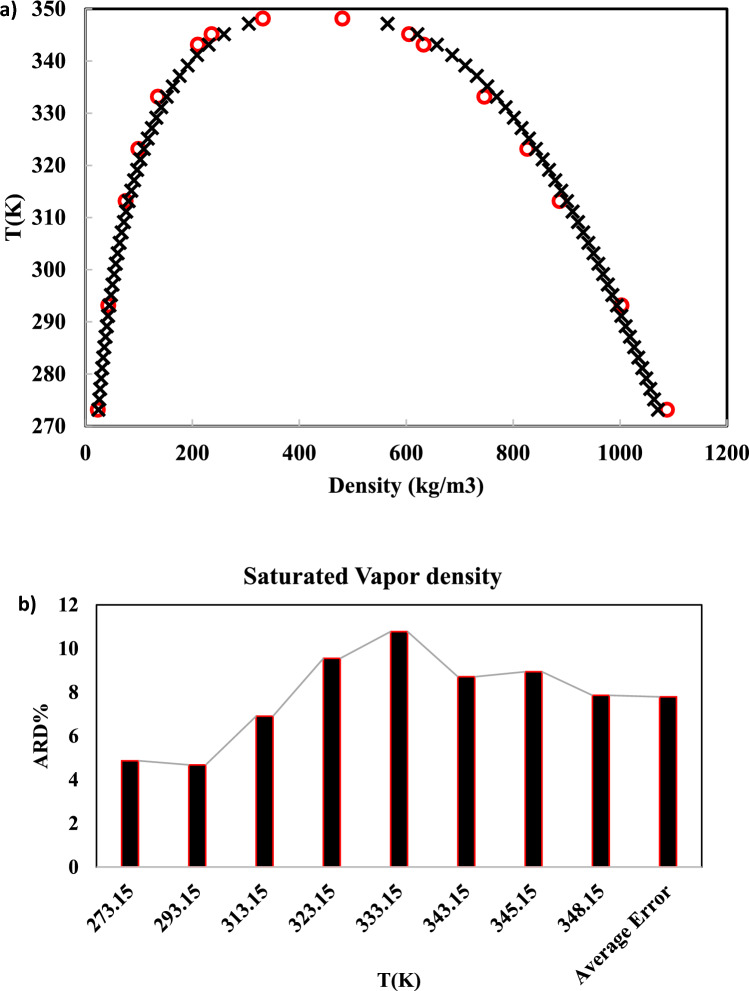
(**a**) Computed saturated liquid and vapor densities of R-454B (○) and REFPROP (×). (**b**) Error bar of saturated vapor density.

One of the main properties of refrigerants is vapor and liquid densities. In this study, the saturated liquid and vapor densities of R-454B at five temperatures have been simulated and compared to REFPROP data. Similar to vapor pressure simulations, the saturated densities have been simulated using the Materials Studio modeling program. In Table 4 and Fig. 3, the results of the simulation and the ARD% have been reported and compared to REFPROP data.

**Table 4:** Simulated saturated liquid and vapor densities of R-454B.

T(K)	${\rho }_{v}^{sat}$(kg/m^3^)		${\rho }_{l}^{sat}$(kg/m^3^)		ARD%	
	Simulated	REFPROP	Simulated	REFPROP	${\rho }_{v}^{sat}$	${\rho }_{l}^{sat}$
273.15	23.14 (± 0.64)	24.32	1087.73 (± 12.1)	1071.01	4.87	1.56
293.15	42.79 (± 0.121)	44.89	1002.85 (± 14.3)	994.05	4.68	0.88
313.15	75.21 (± 1.42)	80.79	887.052 (± 14.6)	900.75	6.91	1.52
323.15	98.87 (± 0.38)	109.31	826.62 (± 10.2)	842.75	9.55	1.91
333.15	135.54 (± 1.67)	151.9 + 2	746.14 (± 11.1)	769.37	10.78	3.01
343.15	210.36 (± 3.24)	230.44	632.61 (± 11.3)	657.23	8.71	3.74
345.15	236.16 (± 2.61)	259.19	605.63 (± 12.2)	620.91	8.94	2.46
348.15	332.30 (± 3.74)	360.35	480.53 (± 11.5)	494.92	7.86	2.89
Average error					**7.79**	**2.25**

Significant values are in bold.

As shown in Table 4, the vapor densities and saturated liquid have been simulated satisfactory. The average ARD% values of liquid densities and saturated vapor have been obtained 7.79% and 2.25%, respectively. Figure 3a demonstrates that the saturated densities of liquid and vapor phases simulated by MD are in good agreement with the REFPROP data. As well, the error bar of saturated vapor density has been depicted in Fig. 3b.

In the MD simulation, the C_p_ measures how the enthalpy responds to an isobaric change^36^:

4 $${C}_{p}={\left(\frac{\partial H}{\partial T}\right)}_{P}=\frac{\partial {({E}_{k}+U+pV)}^{2}}{{k}_{B}{T}^{2}}$$

where *E*_*k*_*, H, U, V, p, T,* and *k*_*B*_ refer to the kinetic energy, enthalpy, potential energy, volume, pressure, temperature and Boltzmann constant, respectively. In Table 5, the liquid specific heat capacity of R-454B has been reported and compared to REFPROP data.

**Table 5:** Specific heat capacity of R-454B.

T(K)	Data points	P(MPa)	${C}_{P}^{Liq}$(kJ/kg K)		ARD (%)
			Simulated	REFPROP	
273.15	1	1	1.756 (± 0.23)	1.613	8.87
	2	2	1.743 (± 0.21)	1.601	8.68
	3	3	1.727 (± 0.32)	1.589	8.42
	4	4	1.712 (± 0.11)	1.579	8.67
	5	5	1.705 (± 0.15)	1.569	6.75
283.15	6	2	1.771 (± 0.23)	1.659	7.31
	7	3	1.762 (± 0.25)	1.642	6.76
	8	4	1.738 (± 0.24)	1.628	7.19
	9	5	1.730 (± 0.18)	1.614	7.83
293.15	10	2	1.874 (± 0.31)	1.738	7.94
	11	3	1.849 (± 0.28)	1.713	8.04
	12	4	1.827 (± 0.28)	1.691	8.13
	13	5	1.808 (± 0.24)	1.672	7.38
298.15	14	2	1.921 (± 0.21)	1.789	7.17
	15	3	1.883 (± 0.19)	1.757	7.51
	16	4	1.861 (± 0.16)	1.731	7.80
	17	5	1.839 (± 0.22)	1.706	6.85
303.15	18	2	1.981 (± 0.15)	1.854	6.68
	19	3	1.932 (± 0.14)	1.811	7.04
	20	4	1.901 (± 0.31)	1.776	6.93
	21	5	1.867 (± 0.34)	1.746	8.87
Average error	**22**				**7.66**

Significant values are in bold.

**Fig. 4:**
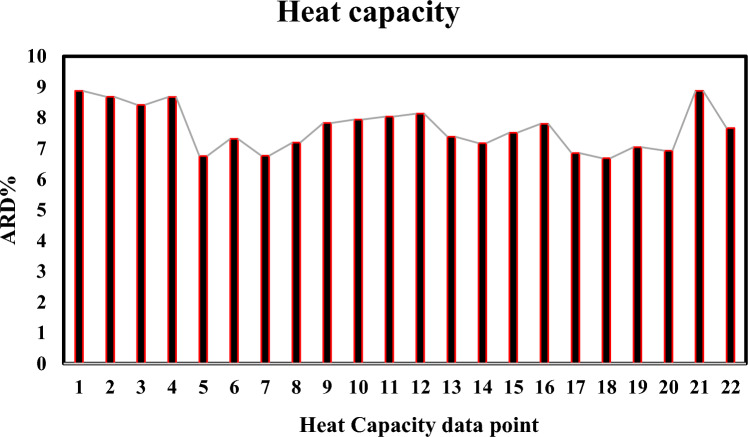
The error bar of specific heat capacity of R-454B.The x-axis refers to data points listed in Table 5.

As shown in Table 5, the average ARD% value of liquid heat capacity of R-454B at five temperatures have been obtained 7.66%. In Fig. 4, the error bar of specific heat capacity has been depicted.

**Fig. 5:**
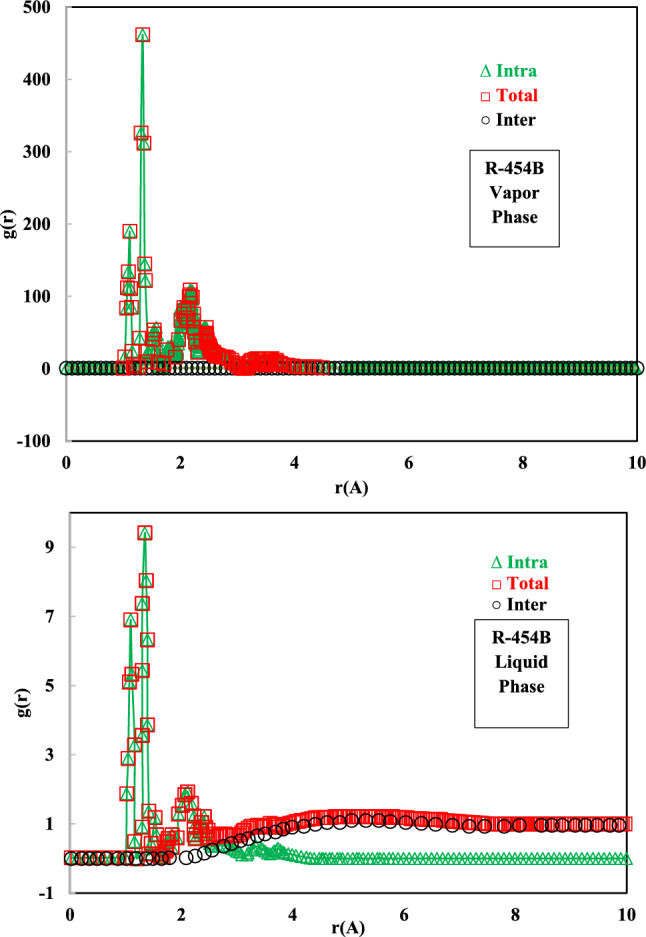
Radial distribution functions (RDF) of R-454B.

The radial distribution function (RDF) describes how the density of atoms varies as a function of distance from a reference atom. In simpler terms, RDF shows the probability of finding another atom at a certain distance *r* away from a given atom. It’s particularly useful for characterizing the structure of disordered systems like liquids and amorphous solids, where long-range order is absent. Peaks in the RDF indicate distances where there is a higher probability of finding other particles. For liquids, the first peak corresponds to the average distance of the nearest neighbors. Subsequent peaks represent distances to second nearest neighbors. Valleys indicate distances where there is a lower probability of finding particles. At large distances, the RDF typically approaches 1, indicating that the probability of finding a particle is uniform. This reflects the lack of long-range order in liquids and gases. The RDF influences the energy, entropy, and other thermodynamic properties of a fluid. These are related to the potential energy of the system as well as the interaction between molecules. Changes in the RDF can be used to characterize phase transitions, such as melting and boiling, especially as it illustrates the transition from a short-range order in liquids to a more long-range order in solids. The shape and position of RDF peaks provide insight into the attractive and repulsive forces between refrigerant molecules. This is critical for understanding intermolecular interactions, and predicting the behavior of a refrigerant in different environments. For refrigerant mixtures, the RDF can reveal how the different components are arranged and how they interact with each other. Experimentally, the RDF is determined through scattering techniques (X-ray or neutron diffraction) which might be difficult in certain cases. Molecular dynamics or Monte Carlo simulations are often used to calculate the RDF of refrigerants. These simulations require accurate intermolecular potential models, especially for the complex molecular structure of modern refrigerants. Specific RDF data for refrigerants might not always be readily available in public databases. It may require dedicated experimental or computational studies. The RDF is defined as follows^36^:

5 $$\text{g}\left(r\right)=\frac{dN}{\rho 4\pi {r}^{2}dr}$$

where *r* refers to the distance between two particles. The simulated RDFs in the gas and liquid phases have been shown in Fig. 5.

Zeng et al. showed that the peaks of RDF curve within 3.5 Ǻ come from hydrogen bond and chemical bond. As well, the peaks outside 3.5 Ǻ come from the Coulomb force and van der Waals force^37^. As shown in Fig. 5, the first highest peak of RDF for the R-454B vapor phase is appeared at about 1.09 Å. The first peak indicates the formation of the C–H bond. The second highest peak was at about 1.35 Å, produced by C–F and C=C bonds. The third peak refers to C–C bond at about 1.51 Å. The fourth peak is at about 2.1 Å which is close to the length of H–F bond. After about 3.5 Ǻ, RDF decreases to zero. As shown in Fig. 5, the positions of the peaks in the RDFs in gas and liquid phases are found to have no changes. These results show that the R-454B molecules were not altered during the condensation procedure. Larger values of higher peaks show the stronger development of C–H and C–F pairs in the vapor phase. On the other hand, when the ratio of the local density to the average density is high, and the average density of the simulation is low, the peak in RDF is high. The peak in RDF tends to decrease when the average density increases. A refrigerant with a higher critical temperature will likely exhibit RDF peaks with higher heights and narrower widths at shorter distances compared to a refrigerant with a lower critical temperature. This reflects stronger and more directional intermolecular attractions that lead to a more ordered and compact liquid structure. Weaker interactions result in broader, lower peaks, indicating less precise and less frequent interactions at the average distances corresponding to those peaks. As shown in Fig. 5, the first highest peak (indicates the formation of the C–H bond) and the second highest peak (indicates the formation of the C–F and C=C bonds), show higher heights and narrower widths at shorter distances. While peak heights and widths are important, the positions of the peaks also matter. If the primary peak in the RDF of the refrigerant with a higher critical temperature is significantly closer to the molecular center of mass, it suggests a more tightly packed structure. A larger distance between molecules at the peak position in the lower critical temperature refrigerant indicates weaker attractions and a less tightly bound liquid phase. The presence of multiple distinct peaks at different distances can provide clues about different types of interactions. For instance, a refrigerant with a higher critical temperature might exhibit sharper peaks indicating more defined interactions at particular distances, possibly due to specific bonding or steric effects. A refrigerant with a lower critical temperature might have broader or less defined peaks, signifying weaker and less specific interactions. The area under the RDF peak is related to the coordination number, the average number of neighboring molecules at a given distance. A refrigerant with a higher critical temperature will likely have a larger coordination number, signifying a denser liquid phase with more neighbors at each molecule, again reflecting stronger interactions. The overall shape of the RDF can also provide insight. A more “peaked” and structured RDF often correlates with stronger intermolecular forces and a higher critical temperature. A more diffuse and less structured RDF suggests weaker interactions.

Shifts in peak positions in the self-RDFs of the blend compared to the pure components can indicate changes in molecular packing due to the presence of the other components. Increased peak heights in the blend RDFs compared to the pure components suggest stronger self-association within the blend. Lower peak heights suggest disruption of self-association. As depicted in Fig. 5, RDF value of the second peak of R-454B blend in vapor phase is about 480, while the corresponding RDF of pure R-1234yf was obtained about 310^4^. RDFs can provide insights into vapor–liquid equilibrium (VLE) and liquid–liquid equilibrium (LLE). Strong attractions between different components (high cross-RDF peaks) generally lead to better miscibility and a more stable liquid phase. On the other hand, understanding the interactions between components in a refrigerant blend can help optimize its performance in terms of cooling capacity, energy efficiency, and environmental impact. RDFs can be linked to transport properties like viscosity and thermal conductivity. Stronger intermolecular forces lead to higher viscosity. Consequently, by carefully comparing and interpreting the RDFs of different refrigerant blends, a deep understanding of their molecular-level interactions can be gained, which ultimately determines their macroscopic properties and performance.

In this study, to get some insight into the vapor condensation process of R-454B on a micro level, density and the potential energy variations during condensation processes were simulated an presented in Supplementary Material.

### Thermodynamic properties prediction

The MD simulations were utilized to estimate the phase equilibrium calculations (vapor pressure and density), and the specific heat capacity of R-454B at various pressures and temperatures. The MD results were compared to REFPROP data to check the evaluate the MD simulation results. One of the main disadvantages of the MD simulations is time-consuming of simulations. In this regard, a robust thermodynamic model is a good alternative to predict the thermophysical properties of refrigerants over a wide range of temperatures and pressures. The main challenge of an EoS (especially SAFT-based models) is the estimation of model parameters. The SAFT-based model parameters should be obtained using experimental data. In the absence of experimental data for a new fluid, it is very difficult to obtain model parameters accurately. In this regard, the simulation data can be used to adjust the model parameters. In this work, the PC-SAFT EoS model has been used to predict the second-order derivative properties of R-454B. The R-454B is considered as a pseudo-component, and the model parameters are optimized using the obtained simulation data in this study.

The PC-SAFT stands for “Perturbed-Chain Statistical Associating Fluid Theory”. It’s a sophisticated EoS used to predict the thermodynamic properties of fluids, particularly complex fluids like polymers, associating fluids (e.g., those that form hydrogen bonds), and mixtures. It’s based on statistical mechanics and perturbation theory. PC-SAFT is based on a more realistic physical model of fluids, taking into account chain length, segment size, and intermolecular interactions. This makes it more reliable for extrapolation and for predicting the behavior of mixtures. The PC-SAFT EoS expresses the residual Helmholtz energy as a sum of contributions^38^:

6 $$\frac{{A}^{res}}{N{k}_{B}T}=\frac{{A}^{hc}}{N{k}_{B}T}+\frac{{A}^{disp}}{N{k}_{B}T}$$

where *res, disp, hc* are residual, dispersion, and hard chain contributions, respectively. Hard-chain contribution (reference fluid) accounts for the repulsive interactions between the segments of the chain. Dispersion contribution (perturbation term) accounts for the attractive (dispersive) interactions between the segments^38^:

7 $$\frac{{A}^{hc}}{N{k}_{B}T}=\overline{m}\frac{{A }^{hs}}{N{k}_{B}T}-\sum_{i}{x}_{i}\left({m}_{i}-1\right)\text{ln}{g}_{ii}^{hs}({\sigma }_{ii})$$

8 $$\frac{{A}^{hs}}{N{k}_{B}T}=\frac{1}{{\xi }_{0}}\left[\frac{3{\xi }_{1}{\xi }_{2}}{(1-{\xi }_{3})}+\frac{{\xi }_{2}^{3}}{{\xi }_{3}{(1-{\xi }_{3})}^{2}}+\left(\frac{{\xi }_{2}^{3}}{{\xi }_{3}^{2}}-{\xi }_{0}\right)\text{ln}(1-{\xi }_{3})\right]$$

9 $$\frac{{A}^{disp}}{N{k}_{B}T}=-2\pi \rho {I}_{1}\left(\eta ,\overline{m }\right)\overline{{m }^{2}\epsilon {\sigma }^{3}}-\pi \rho \overline{m}{C }_{1}{I}_{2}(\eta ,\overline{m })\overline{{m }^{2}{\epsilon }^{2}{\sigma }^{3}}$$

The fugacity coefficient is a crucial thermodynamic property that accounts for the non-ideality of real gases and liquids. Calculating fugacity coefficients using an equation of state is a fundamental task in chemical engineering thermodynamics. The specific formula and complexity depend on the chosen EoS. Cubic EoSs offer a good balance between simplicity and accuracy for many applications, while more complex EoS like PC-SAFT are needed for highly non-ideal systems. The fugacity coefficient of component *i* is given by:

10 $$\text{ln}{\varphi }_{i}=\frac{{\mu }_{i}^{res}}{{k}_{B}T}-\text{ln}Z$$

11 $$Z=1+\rho {\left(\frac{\partial \left({A}^{res}/N{k}_{B}T\right)}{\partial \rho }\right)}_{T,n}$$

12 $${\mu }_{i}^{res}={\left[\frac{\partial \left(n{a}^{res}\right)}{{\partial n}_{i}}\right]}_{T,V,{n}_{j\ne i}}={\left[\frac{\partial \left(\rho {a}^{res}\right)}{{\partial \rho }_{i}}\right]}_{T,{\rho }_{j\ne i}}$$

13 $${\mu }_{i}^{res}={\mu }_{i}^{hc}+{\mu }_{i}^{disp}$$

${\mu }_{i}$ is the chemical potential and a^res^ refers to the residual molar Helmholtz free energy. The second derivative properties such as heat capacity, speed of sound, and Joule–Thomson coefficient can be calculated as follows:

14 $${C}_{v}^{res}\left(T,V,n\right)=-R{T}^{2}{\left(\frac{{\partial }^{2}\left({A}^{res}/{k}_{B}T\right)}{\partial {T}^{2}}\right)}_{v,n}-2RT{\left(\frac{\partial \left({A}^{res}/{k}_{B}T\right)}{\partial T}\right)}_{v,n}$$

15 $${C}_{p}^{res}\left(T,V,n\right)={C}_{v}^{res}\left(T,V,n\right)-\frac{T{{\left(\frac{\partial P}{\partial T}\right)}_{V,n}}^{2}}{{\left(\frac{\partial P}{\partial V}\right)}_{T,n}}$$

16 $${C}_{p}^{res}\left(T,V,n\right)={C}_{P}\left(T,V,n\right)-{C}_{P}^{Ideal gas}\left(T\right)$$

17 $$U=\sqrt{-\frac{{V}^{2}}{Mw}\frac{{C}_{p}}{{C}_{v}}{\left(\frac{\partial P}{\partial V}\right)}_{T}}$$

18 $${\mu }_{JT}={\left(\frac{\partial T}{\partial p}\right)}_{H}=\frac{1}{{C}_{p}}\left(T{\left(\frac{dV}{dT}\right)}_{p}-V\right)$$

where ${C}_{v}^{res}$, ${C}_{p}^{res}$, ${\mu }_{JT}$, and $U$, are residual isochoric heat capacity, isobaric heat capacity, Joule–Thomson, and speed of sound, respectively.

The PC-SAFT model parameters of R-454B as a pseudo-component have been adjusted using the following objective function (OF):

19 $$OF=\sum_{i}{\left[\frac{{\rho }_{i}^{ sat,sim}-{\rho }_{i}^{ sat,calc}}{{\rho }_{i}^{ sat,sim}}\right]}^{2}+\sum_{i}{\left[\frac{{p}_{i}^{ sat,sim}-{p}_{i}^{ sat,calc}}{{p}_{i}^{ sat,sim}}\right]}^{2}+0.2\sum_{i}{\left[\frac{{C}_{p}^{ sim}-{C}_{p}^{ calc}}{{C}_{p}^{ sim}}\right]}^{2}$$

where *sat*, *sim*, and *calc* refer to saturation, simulation, and calculated data. As shown in Eq. (19) a weight value (0.2) has been considered for isobaric heat capacity OF. The weight value varies between 0 and 1. For density and vapor pressure maximum weight values were selected, while in the case of isobaric heat capacity, the optimum value was obtained 0.2. The higher values increase the error values of saturated density and vapor pressure dramatically. In this work the optimum value was obtained between 0.2 and 0.3. In Table 6 the PC-SAFT model parameters and the ARD% values have been presented.

**Table 6:** PC-SAFT EoS parameters in two scenarios.

Pseudo-components	Parameters			ARD (%)			T range (K)	Ref
	*m(–)*	$\sigma (\dot{\text{A}})$	$\varepsilon /{k}_{B}(\text{K})$	P^sat^	${\rho }^{sat}$	Cp		
R-454B	2.7444	2.8683	172.3598	0.036	1.32	4.3	273.15–333.15	This work

As shown in Table 6, the R-454B has been considered as a pseudo-component, and its parameters have been optimized using the saturated liquid density, isobaric heat capacity, and vapor pressure simulation data. The average ARD% values of saturated pressure, saturated density, and isobaric heat capacity have been obtained 0.036%, 1.32%, and 4.3%, respectively. The sensitivity analysis of PC-SAFT parameters has been presented in Supplementary Material. This result indicates that, the segment energy (*ε)* parameter is a crucial variable for vapor pressure calculations. On the other hand, the segment diameter (*σ)* parameter is a crucial variable for density predictions. Thermodynamic properties of components can be divided into two categories; first-order derivative and second-order derivative properties. Vapor pressure and density are first-order derivative properties. The isobaric/isochoric heat capacity, speed of sound and Joule–Thomson coefficient are second-order derivative thermodynamic properties. These second-order properties give us information about the system’s response to changes in temperature and pressure. They can be important in a variety of applications including in the behavior of fluids in extreme conditions and in understanding the nature of phase transitions. If there are errors in calculating the first derivatives, these errors will propagate and get amplified when calculating the second derivatives, which can lead to large prediction uncertainties. Second-order derivatives are more sensitive to the underlying intermolecular interactions and how these interactions are influenced by temperature and pressure. Predicting second-order derivative thermodynamic properties is indeed complex due to their high sensitivity, data limitations, modeling intricacies, and computational demands. Techniques like MD and Monte Carlo (MC) can provide detailed information and predictions of both first and second-order properties. However, they are computationally expensive. The equations of state are still common for predicting thermodynamic properties. In this regard, a SAFT-based EoS such as the PC-SAFT model can be utilized to predict the second-order derivative thermodynamic properties of R-454B over a wide range of pressures and temperatures.

**Fig. 6:**
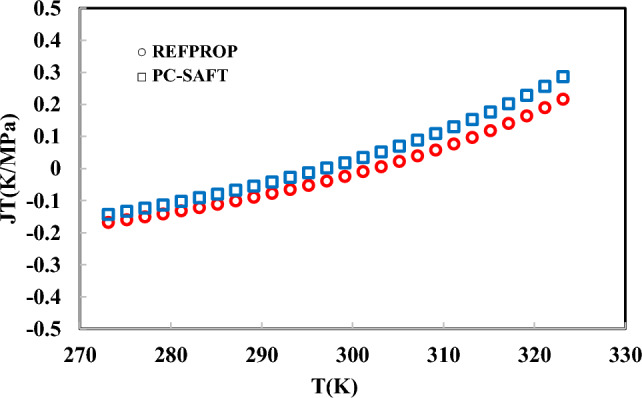
Joule–Thomson prediction using PC-SAFT EoS at 10 MPa.

**Fig. 7:**
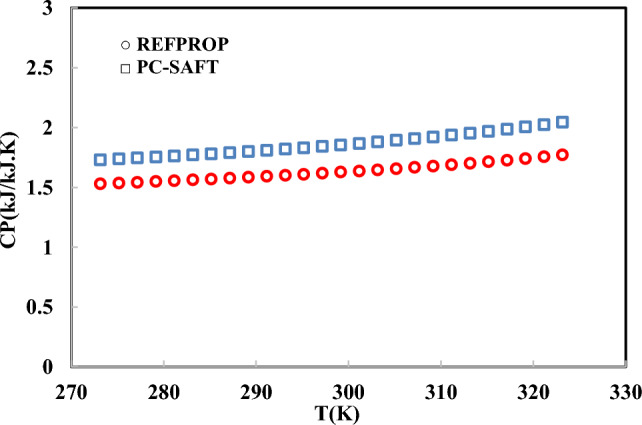
Isobaric heat capacity prediction using PC-SAFT EoS at 10 MPa.

**Fig. 8:**
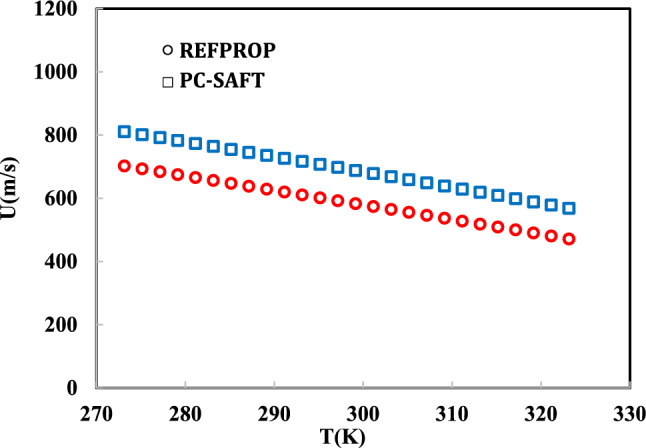
Speed of sound prediction using PC-SAFT EoS at 10 MPa.

The model performance has been evaluated using the speed of sound, specific heat capacity, and Joule–Thomson coefficient over a wide range of pressures and temperatures. In Figs. 6, 7 and 8, the speed of sound, specific heat capacity, and Joule–Thomson coefficient of R-454B have been precited using the PC-SAFT EoS and compared to REFPROP data.

As shown in Figs. 6, 7, 8, the isobaric heat capacity, Joule–Thomson coefficient, and speed of sound have been predicted using the obtained parameters in Table 6. As mentioned, the model parameters have been optimized using simulation data based on Eq. (19). In Fig. 6, the Joule–Thomson coefficient of R-454B has been precited between 273.15 and 323.15 K, satisfactory. In Figs. 6, 7 and 8, the aforementioned thermodynamic properties were predicted at high pressure (100 bar). A robust EoS must be able to predict the first- and second-order derivative thermodynamic properties of R-454B in liquid and vapor phases. In this regard, in Tables 7 and 8, the results of the PC-SAFT EoS have been evaluated and compared to REFPROP data in liquid and vapor phases.

**Table 7:** Prediction of isochoric and isobaric heat capacity, speed of sound, and Joule–Thomson coefficient of R-454B at 0.1 MPa and 200–330 K.

T (K)	P (MPa)	Phase	Cv (kJ/kg K)		Cp (kJ/kg K)		U (m/s)		JT (K/MPa)	
			REFPROP	PC-SAFT	REFPROP	PC-SAFT	REFPROP	PC-SAFT	REFPROP	PC-SAFT
200	0.1	Liquid	0.88	0.96	1.4	1.6	996.38	1103.1	− 0.32	− 0.31
210	0.1	Liquid	0.88	0.96	1.42	1.61	945.14	1055.8	− 0.30	− 0.30
220	0.1	Liquid	0.88	0.96	1.44	1.63	893.89	1008.1	− 0.28	− 0.29
230	0.1	Vapor	0.66	0.63	0.83	0.90	188.4	204.98	61.25	21.00
240	0.1	Vapor	0.66	0.64	0.82	0.92	192.79	208.95	50.94	19.16
250	0.1	Vapor	0.66	0.66	0.82	0.94	196.93	212.80	43.2	17.54
260	0.1	Vapor	0.67	0.68	0.82	0.95	200.89	216.52	37.11	16.11
270	0.1	Vapor	0.68	0.70	0.83	0.97	204.7	220.14	32.2	14.85
280	0.1	Vapor	0.7	0.71	0.84	0.99	208.38	223.67	28.19	13.72
290	0.1	Vapor	0.71	0.73	0.85	1.00	211.95	227.10	24.86	12.70
300	0.1	Vapor	0.72	0.75	0.87	1.02	215.42	230.46	22.09	11.79
310	0.1	Vapor	0.74	0.76	0.88	1.04	218.81	233.74	19.75	10.97
320	0.1	Vapor	0.75	0.78	0.89	1.05	222.11	236.96	17.76	10.23
330	0.1	Vapor	0.77	0.80	0.91	1.07	225.35	240.11	16.06	9.55
340	0.1	Vapor	0.78	0.82	0.924	1.09	228.53	243.20	14.59	8.93
345	0.1	Vapor	0.79	0.83	0.932	1.09	230.09	244.72	13.93	8.65
348	0.1	Vapor	0.79	0.83	0.936	1.10	231.02	245.63	13.56	8.48
350	0.1	Vapor	0.80	0.83	0.939	1.10	231.64	246.23	13.32	8.37
360	0.1	Vapor	0.81	0.85	0.95	1.12	234.70	249.22	12.21	7.86
370	0.1	Vapor	0.83	0.87	0.97	1.14	237.71	252.15	11.23	7.38
380	0.1	Vapor	0.84	0.89	0.98	1.15	240.68	255.04	10.3	6.95
400	0.1	Vapor	0.88	0.92	1.01	1.19	246.48	260.68	8.90	6.18

**Table 8:** Prediction of isochoric and isobaric heat capacity, speed of sound, and Joule–Thomson coefficient of R-454B at 1 MPa and 200 K to 255 K.

T (K)	P (MPa)	Phase	Cv (kJ/kg K)		Cp (kJ/kg K)		U (m/s)		JT (K/MPa)	
			REFPROP	PC-SAFT	REFPROP	PC-SAFT	REFPROP	PC-SAFT	REFPROP	PC-SAFT
200	1	Liquid	0.88	0.957	1.40	1.60	1000.80	1106.6	− 0.33	− 0.32
205	1	Liquid	0.88	0.957	1.41	1.60	975.34	1083.1	− 0.32	− 0.31
210	1	Liquid	0.88	0.957	1.42	1.61	949.91	1059.5	− 0.31	− 0.30
215	1	Liquid	0.88	0.956	1.43	1.62	924.49	1035.9	− 0.30	− 0.30
220	1	Liquid	0.88	0.956	1.44	1.409	899.08	1012.2	− 0.28	− 0.29
225	1	Liquid	0.88	0.956	1.45	1.289	873.66	988.4	− 0.27	− 0.28
230	1	Liquid	0.88	0.956	1.46	1.222	848.21	964.5	− 0.26	− 0.27
235	1	Liquid	0.89	0.956	1.47	1.179	822.71	940.5	− 0.24	− 0.26
240	1	Liquid	0.89	0.956	1.48	1.150	797.13	916.3	− 0.22	− 0.24
245	1	Liquid	0.89	0.956	1.50	1.130	771.43	892.0	− 0.20	− 0.23
250	1	Liquid	0.90	0.957	1.51	1.116	745.59	867.4	− 0.18	− 0.21
260	1	Liquid	0.90	0.957	1.55	1.099	693.25	817.5	− 0.13	− 0.17
270	1	Liquid	0.91	0.957	1.60	1.091	639.62	766.3	− 0.06	− 0.12
280	1	Liquid	0.92	0.957	1.66	1.090	583.96	713.4	0.02	− 0.05
290	1	Liquid	0.84	0.957	1.17	1.093	189.72	658.0	25.16	0.03
300	1	Liquid	0.83	0.957	1.11	1.099	196.08	599.1	22.31	0.16
310	1	Liquid	0.82	0.957	1.07	1.106	201.74	535.1	19.93	0.34
320	1	Liquid	0.82	0.955	1.05	1.116	206.91	462.1	17.91	0.65
330	1	Liquid	0.82	0.949	1.04	1.126	211.71	367.1	16.18	1.37
340	1	Liquid	0.83	0.939	1.04	1.138	216.22	249.5	14.69	3.65
345	1	Liquid	0.83	0.943	1.04	1.144	218.39	249.3	14.02	3.61
348	1	Liquid	0.84	0.946	1.04	1.148	219.66	249.2	13.64	3.59
350	1	Liquid	0.84	0.948	1.04	1.150	220.50	249.2	13.39	3.57
360	1	Liquid	0.85	0.958	1.04	1.163	224.57	249.0	12.25	3.50
370	1	Liquid	0.86	0.968	1.04	1.177	228.48	249.2	11.25	3.42
380	1	Liquid	0.87	0.979	1.05	1.191	232.24	249.7	10.37	3.34
400	1	Liquid	0.90	1.002	1.07	1.220	239.39	251.8	8.88	3.16

The results show that the obtained PC-SAFT model parameters (which were adjusted using the simulation results) can predict the second-order derivative thermodynamic properties of R-454B satisfactory. As shown in Table 7, the Joule–Thomson coefficient in the vapor phase gives a higher deviation compared to the liquid phase. In Table 8, the liquid phase Joule–Thomson coefficient was well predicted. The speed of sound of the vapor and liquid phases can be well predicted using the PC-SAFT EoS.

The results show that the suggested method for the prediction of thermodynamic properties of R-454B refrigerant can be extended to various pure and mixed systems. This approach is useful for the pre-design of new refrigerants in the absence of experimental data. In this approach, the MD simulation can be performed to calculate the density, vapor pressure, and one second-order derivative properties for new refrigerant (or refrigerant mixtures). Then the SAFT-based model parameters will be optimized using the aforementioned simulated properties. Finally, the obtained parameters can be utilized to predict the phase equilibrium calculations and thermophysical properties of the system.

## Conclusion

In this study, the molecular dynamic (MD) simulation was performed to estimate the thermodynamic properties of R-454B refrigerant. The vapor pressure, saturated liquid density, and isobaric heat capacity of R-454B were simulated at temperatures ranging from 273.15 to 348.15 K. The obtained results were in good agreement with REFPROP data. The average ARD% value of simulated vapor pressure, saturated vapor and saturated liquid densities were obtained 1.79%, 7.79%, and 2.25%, respectively. As well, the average ARD% value of isobaric heat capacity was obtained 7.66%. In this work, the Radial Distribution Function (RDF) of R-454B in vapor and liquid phases has been calculated. The positions of the peaks in the RDFs in gas and liquid phases are found to have no changes. These results show that the R-454B molecules were not altered during the condensation procedure. The MD simulation needs extensive computational time, while a thermodynamic model such as SAFT-based EoS is much faster than MD. In this regard, a new methodology was proposed to predict the thermophysical properties and phase equilibrium calculations of R-454B over a wide range of pressures and temperatures. In this regard, the simulated data was used to adjust the PC-SAFT EoS parameter. The second-order derivative thermodynamic properties like isochoric/isobaric heat capacity, speed of sound, and Joule–Thomson coefficient of R-454B over a wide range of pressures and temperatures were predicted using the PC-SAFT EoS, satisfactory. The PC-SAFT EoS results were compared to the REFPROP data. The results were in good agreement with REFPROP data in vapor and liquid phases. This work shows that the proposed methodology for the prediction of thermodynamic properties of R-454B can be extended to various pure and mixed refrigerants. This approach is useful for the pre-design of new refrigerants in the absence of experimental data.

## Supplementary Information

Supplementary Information.

## Data Availability

All data generated or analysed during this study are included in the published articles (that they have been cited) or in the Figures and Tables of this study.
