# Supplementary material for: Prediction of thermophysical properties of R-454B based on molecular dynamic simulation and SAFT-based equation of state
Source: Sci Rep. 2025 Jun 5;15:19832. doi: 10.1038/s41598-025-03928-2 (PMC12141669; doi:10.1038/s41598-025-03928-2)
Supplement: Supplementary file 1 — Supplementary Information. [file 41598_2025_3928_MOESM1_ESM.pdf]

Density and potential energy variations during condensation processes

To get some insight into the vapor condensation process of R-454B on a micro level, density and the potential energy variations during condensation processes were simulated and shown in Figures A1 and A2. The total potential energy of R-454B is the sum of van der Waals energy, electrostatic energy, total valence energy, and total cross-term energy. It can be seen from Figure A2 that the total potential energy, the total cross-term energy and the electrostatic energy are always negative, and the total valence energy is always positive. The total potential energy, the electrostatic energy and the van der Waals energy decrease slowly at the beginning of the condensation

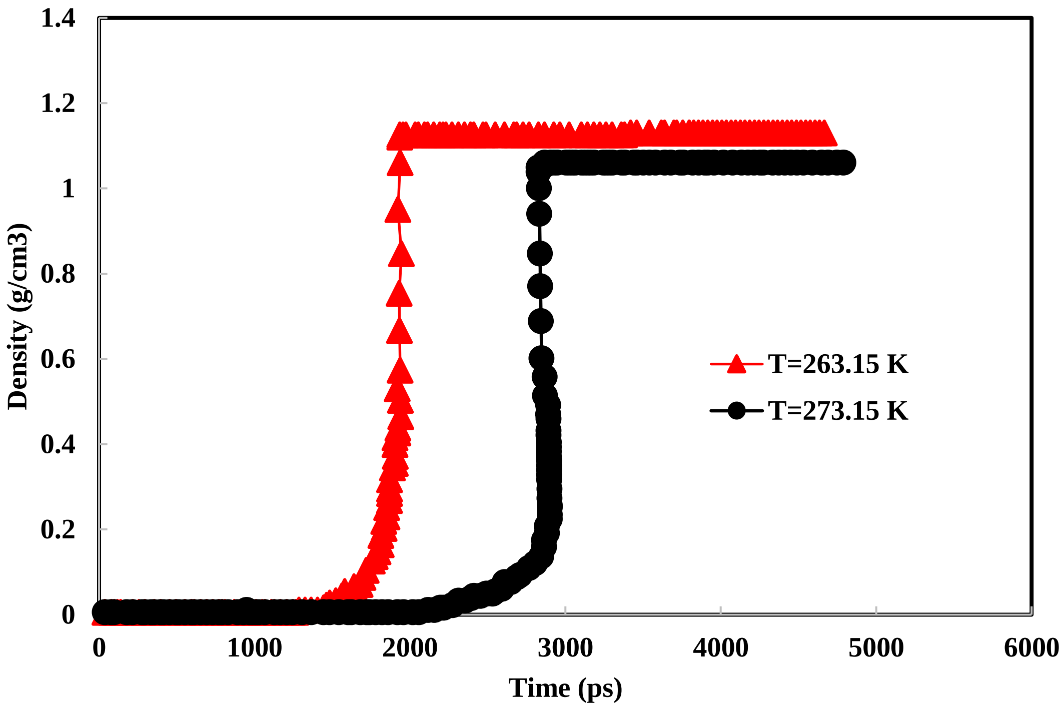

Figure A1. Density change of R-454B during condensation process at 1 MPa

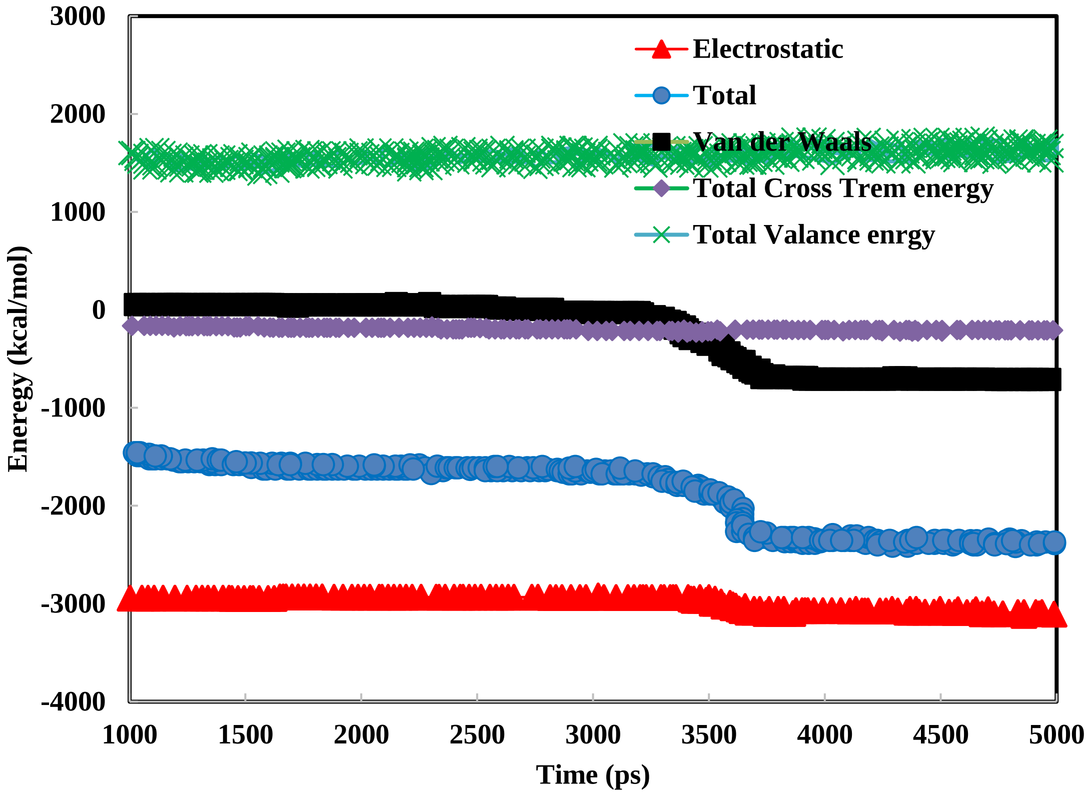

Figure A2. The potential energy of R-454B at 273.15 K and 1 MPa.

It can be seen that the absolute value of the electrostatic energy is much larger than the van der Waals energy, indicating that the electrostatic energy gives the most important contribution to the total potential energy.

Sensitivity analysis of PC-SAFT parameters

Sensitivity analysis of PC-SAFT parameters and their impact on final results, like phase equilibria or thermodynamic properties have been described. This is a crucial step in SAFT modeling to understand which parameters have the most significant influence on predictions. In this work, three model parameters containing segment diameter, segment energy, and segment number were adjusted using experimental vapor pressure, saturated liquid density, and heat capacity data.

- Segment Number (m): Represents the number of segments or “building blocks” that make up a molecule. It’s related to the size and shape of the molecule. Higher ‘m’ usually indicates a larger molecule. Sometimes this is represented as effective chain length.

- Segment Diameter (σ): Represents the effective size or diameter of a segment. It influences the repulsive forces between molecules.
- Dispersion Energy Parameter (ε): Represents the strength of the attractive (dispersive) forces between segments. ‘ε’ is the energy parameter. This is usually temperature dependent.

One-at-a-Time (OAT) method is the simplest method. We vary each parameter individually while keeping the others fixed at their base values. Calculate the change in the output variable (e.g., vapor pressure) for each parameter variation. Plot the results to visualize the sensitivity.

In Figure B1 and B2, the OAT analysis of three adjustable parameters on vapor pressure and saturated liquid density have been illustrated.

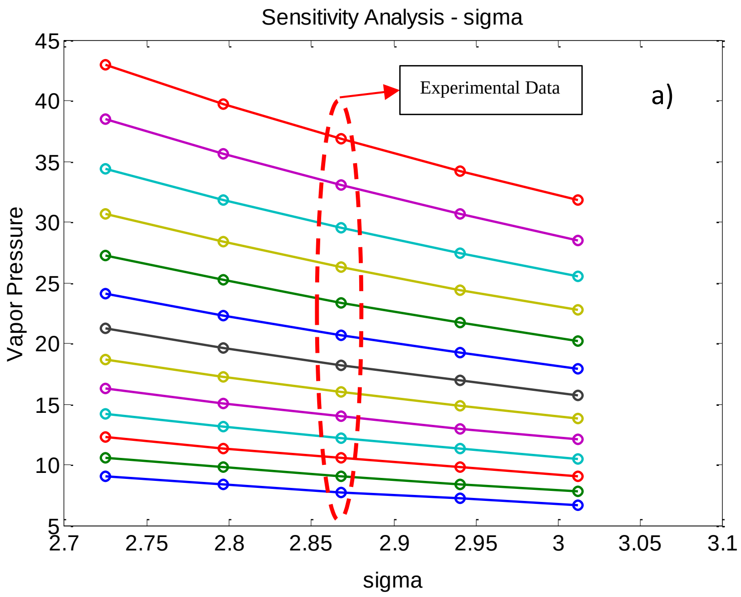

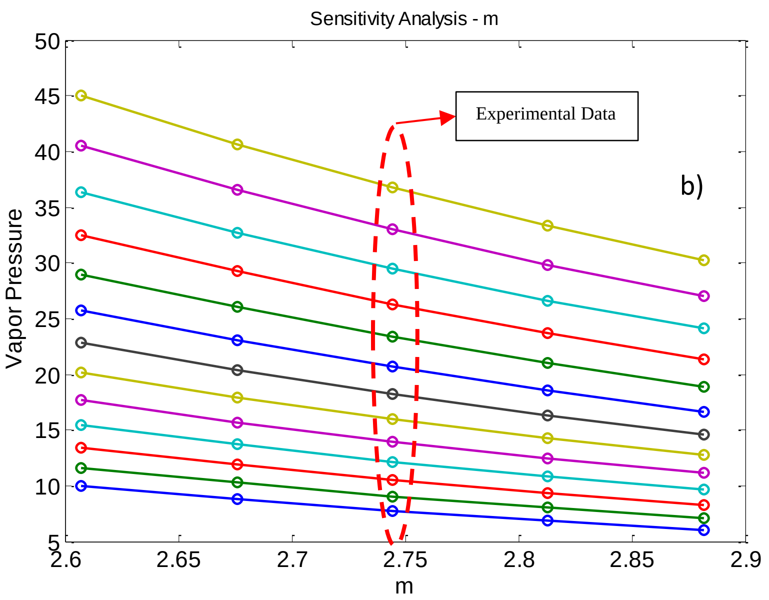

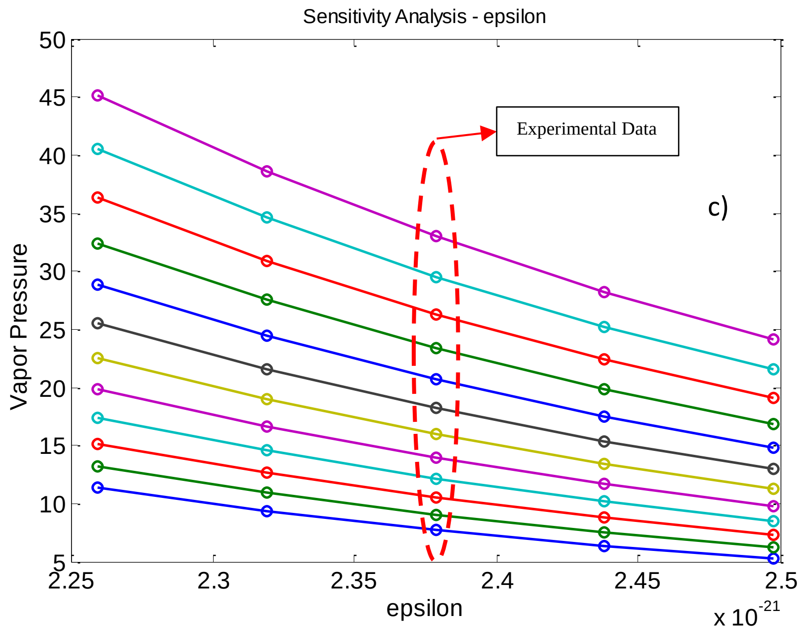

Figure B1. The OAT analysis of PC-SAFT parameters. a) σ vs P^{sat}, b) m vs P^{sat}, c) ε vs P^{sat}.

As shown in Figure B1, the x-axis indicates the PC-SAFT model parameter (m, σ, ε) and y-axis shows the output (predicted vapor pressure). A steep slope at parameters indicates it's a critical variable. If a parameter shows a smaller change or even a plateau, it indicates lower sensitivity.

The slopes of *m*, *σ*, and *ε* have been compared. The results show that, the slopes of σ are lower than *m* and *ε*. On the other hand, the slope of *ε* is higher than *m* and *σ*. This result indicates that, the segment energy (*ε)* parameter is a crucial variable.

In Figure B2., the effect of three model parameters on saturated liquid density calculations have been depicted.

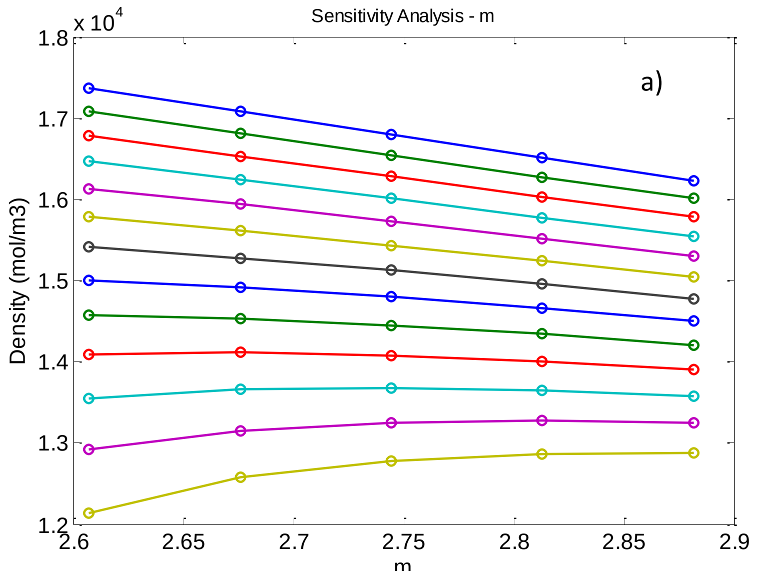

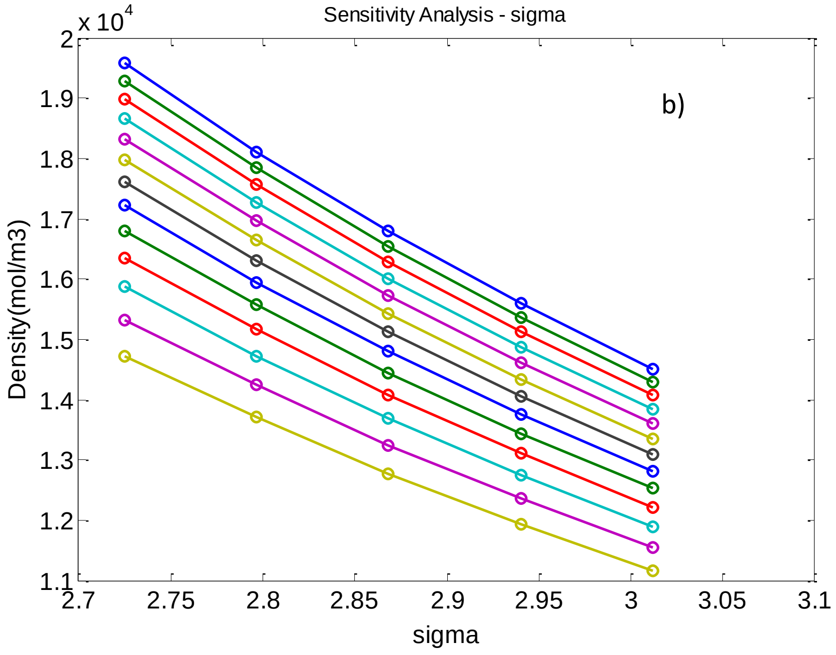

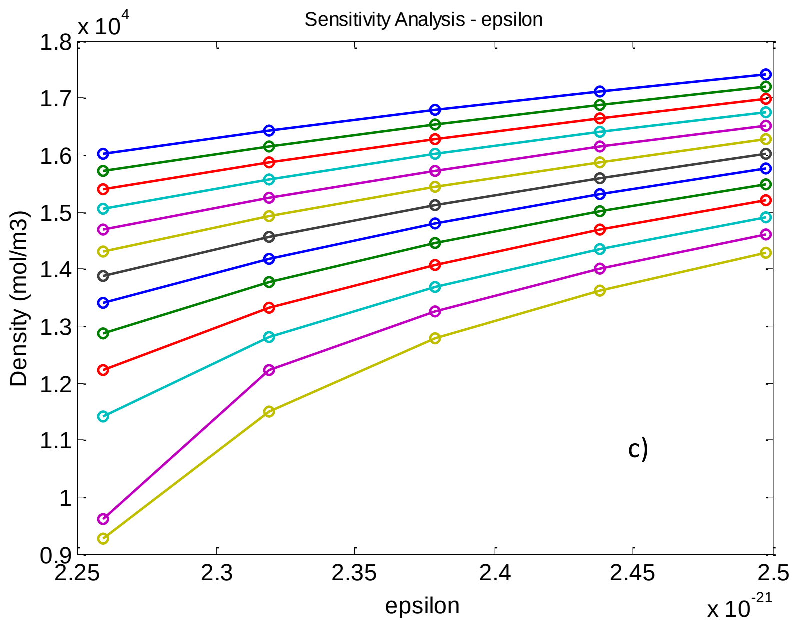

Figure B2. The OAT analysis of PC-SAFT parameters. a) σ vs ρ^{sat}, b) m vs ρ^{sat}, c) ε vs ρ^{sat}.

Similar to Figure B1, the slopes of m, σ, and ε have been compared. The results show that, the slopes of m are lower than σ and ε. On the other hand, the slope of σ is higher than m and ε. This result indicates that, the segment diameter (σ) parameter is a crucial variable for density predictions.
